# The Long-Term Clinical Effectiveness of Ustekinumab in Antitumor Necrosis Factor-Experienced Crohn’s Disease Patients

**DOI:** 10.7759/cureus.28536

**Published:** 2022-08-29

**Authors:** Mansour Altuwaijri, Loai Hakami, Othman Alharbi, Majid Almadi, Suliman Alshankiti, Abdulrahman Aljebreen, Nahla Azzam

**Affiliations:** 1 Division of Gastroenterology, Department of Medicine, College of Medicine, King Khalid University Hospital, King Saud University, Riyadh, SAU; 2 Department of Medicine, King Fahad Central Hospital, Ministry of Health, Jazan, SAU; 3 Department of Medicine, College of Medicine, King Khalid University Hospital, King Saud University, Riyadh, SAU

**Keywords:** predictors, anti-tnf experienced, crohn’s disease, ustekinumab, clinical effectiveness

## Abstract

Background

Crohn’s disease (CD) is a chronic inflammatory bowel disease (IBD) of unknown etiology. Ustekinumab (UST), an interleukin (IL)-12 and IL-23 antibody, has been approved in the recent years to treat IBD, both Crohn’s disease and ulcerative colitis. This study clarifies the long-term effectiveness of ustekinumab (UST) in antitumor necrosis factor (anti-TNF) refractory Crohn’s disease in Middle Eastern patients.

Methods

A retrospective review study, including 30 refractory or medication-intolerant patients with Crohn’s disease, was conducted at a tertiary care center in Riyadh, Saudi Arabia. The patients were started on ustekinumab and followed up for at least 52 weeks. Follow-up was performed on weeks 12, 24, and 52. Data related to demographic and laboratory parameters, the dosing schedule of ustekinumab administration, and the Harvey-Bradshaw index (HBI) were collected. Clinical remission and response rates were assessed. Statistical analysis was performed using SPSS Statistics version 28.0 (IBM Corp., Armonk, NY, USA). A statistical significance threshold of p < 0.05 was adopted.

Results

The mean age of the study subjects was 34.2 ± 17.9 years (95% confidence interval (CI): 27.5-40.9), with a mean disease duration of 10.6 ± 4.9 years (95% CI: 8.8-12.5). Of our cohort, 56.7% failed two biologics during their disease course, and about 20% failed three different biologics. The percentage of patients who used thiopurines was 76.7%, while 6.7% used methotrexate. Concurrent immunomodulators were used by 58.6% of the patients. Corticosteroids were given to 13.3% of the patients. Intravenous induction of UST at 6 mg/kg was used for 90% of the patients, while only 10% used a 260 mg subcutaneous dose. At week 12, 73.3% of the patients had a clinical response, and 66.7% achieved clinical remission. Corticosteroid-free remission, clinical response, and clinical remission showed a decreasing percentage trend between weeks 12 and 24 compared to week 52 where a spike was observed in all aforementioned parameters. The clinical response rate at week 52 was 76.7%. The p-values from cross-tabulation were significant for clinical response and remission when comparing week 12 to weeks 24 and 52.

Conclusion

Ustekinumab presents a safe and effective treatment option in moderate to severe Crohn’s disease patients with previous exposure to multiple biologics.

## Introduction

Crohn’s disease (CD) is a chronic inflammatory bowel disease (IBD) of unknown etiology. Describing the epidemiology of CD is essential for appreciating the public health burden it causes and for planning appropriate health services for people with CD [1-3]. The incidence of Crohn’s disease (CD) has increased worldwide over the past 20 years [4]. The annual CD incidence in Saudi Arabia is estimated to be 0.94 per 100,000 persons per year over these 20 years [5]. However, the overall prevalence of IBD was found to be 26.25 per 100,000 persons [6,7].

Despite the increase in the incidence of CD found among Saudis in recent years, there is very little data published about the characteristics of these patients and the course of the disease in the Kingdom of Saudi Arabia [8-11]. Antitumor necrosis factor (anti-TNF) agents are effective therapies in managing patients with Crohn’s disease [12,13]. Nevertheless, up to one-third of patients are primary nonresponders (PNR), while 25%-40% of patients could develop secondary loss of response (SLR) or intolerance during their treatment [14-16]. Therefore, other emerging biologics with mechanisms different from anti-TNF agents have been developed and approved for CD treatment [17]. Ustekinumab (UST) is a human immunoglobulin G1 (IgG1) kappa monoclonal antibody that blocks interleukin (IL)-12 (IL-12). IL-23 was initially approved for treating psoriasis and active psoriatic arthritis [18]. In 2016, it was approved for the treatment of CD. UST’s trial results (UNITI-1, UNITI-2, and IM-UNITI) positioned this medication well in the armamentarium for CD management [17,19]. It was approved for treating moderate to severe active CD in adults who were intolerant to treatment with corticosteroids or immunomodulators but never failed treatment with tumor necrosis factor (TNF) antagonists or intolerant to therapy with ≥1 TNF antagonists. The real-world outcome of UST treatment is scarce. Furthermore, no long-term results have been reported on the effectiveness and safety of UST from the Middle East. Here, we present a retrospective cohort of CD patients who failed multiple anti-TNF biologics and were treated with UST. The study aimed to evaluate the effectiveness and safety of UST as a treatment for Crohn’s disease in adult patients at a single tertiary care hospital in Riyadh, Saudi Arabia.

## Materials and methods

After appropriate approval from the Ethical Committee of King Saud University Medical City (KSUMC) under Institutional Review Board (IRB) approval number E-11-538, we carried out a retrospective review of adults diagnosed with CD and identified through electronic health records at a tertiary care center in Riyadh, Saudi Arabia. The inclusion criteria were age > 16 years and patients either refractory (clinical or endoscopically) or intolerant to anti-TNFs or anti-TNF and vedolizumab who were started on UST and followed up for at least 52 weeks post-UST. Demographic, clinical, and therapeutic data were also collected, including sex, age, disease duration, severity (at the time of diagnosis), family history, cigarette smoking, perianal disease, disease behavior, previous and concomitant CD medication (including corticosteroids, budesonide, immunomodulators, anti-TNF, and vedolizumab) [20]. Laboratory parameters were measured, including hemoglobin level, erythrocyte sedimentation rate (ESR), and C-reactive protein (CRP) level. The dose and schedule of UST administration and Harvey-Bradshaw index (HBI) [21] were collected. Follow-up data were retrieved at weeks 12, 24, and 52. The initial intravenous (IV) UST dose was weight-based at 6 mg/kg, and the subcutaneous (SC) dose was 260 mg; then, eight weeks after the induction dose, further SC UST was given subcutaneously at eight weeks interval. Dose escalation to every four weeks was allowed as per physicians’ discretion. Clinical remission and response rates were assessed using the HBI, where a clinical response is defined as an HBI decrease of ≥3 and clinical remission as an HBI score of ≤4 points. Glucocorticoid-free remission and adverse events at 12, 24, and 52 weeks and at the end of follow-up were also reported.

Statistical methods

Descriptive statistics were computed for continuous variables, including means, standard deviations (SDs), minimum and maximum values, 95% confidence intervals (CIs) where appropriate, and frequencies for categorical variables. Univariate and multivariate logistic regression analysis was performed to determine the significance of various predictive factors and odds ratio (OR) and their corresponding 95%CIs.

When hypothesis testing was conducted, the paired t-test and Fisher’s exact test, where appropriate, were used. A one-way analysis of variance (ANOVA) was used to test for differences among groups. In our analysis, we used SPSS Statistics version 28.0 (IBM Corp., Armonk, NY, USA). A statistical significance threshold of p = 0.05 was adopted. No attempt at imputation was made for missing data.

## Results

Demographic and clinical characteristics of the study participants

Of the 30 patients in this study, 50% were males. The enrolled patients had a mean age of 34.2 ± 17.9 years (95%CI: 27.5-40.9). The mean disease duration was 10.6 ± 4.9 years (95%CI: 8.8-12.5). There was a positive family history of CD in two (8%) patients. Disease location was ileal in five (16.7%) of the patients and ileocolonic in 20 (66.7%); three (10%) had a colonic disease, and two (6.6) had upper gastrointestinal disease (Table 1).

**Table 1:** Clinical and demographic characteristics of the study participants (n = 30) L1: ileal; L2: colonic; L3: ileocolonic; L4: disease proximal to the terminal ileum (TI)/upper gastrointestinal disease B1: non-stricturing, non-penetrating disease; B2: stricturing disease; B3: penetrating disease BMI: body mass index

Variables	Number (%)	Percentage
Gender		
Male	15	50
Female	15	50
Active smoking	1	3.3
Low BMI < 18.5	10	30
Family history		
Yes	2	6.7
No	23	76.7
At induction		
A1	2	6.7
A2	24	80
A3	3	10
Disease location		
L1	5	16.7
L2	3	10
L3	20	66.7
L4	2	6.6
Disease behavior		
B1	6	20
B2	13	43.3
B3	11	36.7
Extraintestinal manifestations		
None	22	73.3
Arthritis/arthralgia	4	13.3
Skin rashes	2	6.7
Uveitis	1	3.3
Primary sclerosing cholangitis	1	3.3
Previous immunomodulator therapy		
None	2	6.7
Thiopurine	23	76.7
Methotrexate	2	6.7
Previous biologic therapy		
Infliximab	24	80
Adalimumab	20	60
Certolizumab	7	23
Vedolizumab	2	6.7
Concomitant medication		
Immunomodulator	17	56.7
Corticosteroids	4	13.3
Dose of corticosteroids		
10-20 mg	3	1
>20 mg	1	3.3
Method of induction		
Intravenous (6 mg/kg)	27	90
Subcutaneous (260 mg)	3	10
Maintenance dose		
Q 8 weeks	24	80
Dose escalated to Q 4 weeks	6	20

Of the cohort, 43.3% had stricturing disease (B2), 36.7% had penetrating disease (B3), and 20% had non-stricturing, non-penetrating disease (B1). Extraintestinal manifestation was observed in 26.7% of our cohort. Of our cohort, 17 (56.7%) failed two biologics during their disease course, and six (20%) failed three different biologics. Furthermore, 23 (76.7%) patients used thiopurines, while two (6.7%) used methotrexate. Concurrent immunomodulators were used by 17 (58.6%) patients. Corticosteroids were given to four (13.3%) patients; three (10%) received a dose of 10-20 mg and one (3.3%) a dose of >20 mg. Most patients (90%) used the intravenous induction dose at 6 mg/kg, and only 10% used a 260 mg subcutaneous dose.

Clinical efficacy

We calculated clinical response and remission based on the HBI at the end of follow-up compared to baseline (Table 2). At week 12, 23/30 (73.3%) patients had a clinical response (HBI decrease ≥ 3), and 20/30 (66.7%) achieved clinical remission (HBI score ≤ 4 points). Data on HBI at week 12 were missing for two patients with ongoing UST treatment. Of the patients, 76.7% at week 12 and 40% at the end of follow-up were in glucocorticoid-free remission.

Overall, the median ESR was 38 mm/hour (mean: 40.4 + 8.9; interquartile range (IQR): 18-60; 95%CI: 29.5-51.3) at baseline and 46 mm/hour (mean: 32.5 + 13.1; IQR: 19-62; 95%CI: 4.1-60.9) at the end of follow-up; analysis of variance between baseline ESR and final ESR value generated a p-value of 0.7. The median of HBI was 2 (IQR: 0-4) at baseline and 0 (IQR: 0-0) at the end of follow-up. Analysis of variance between HBI at baseline and at 12, 24, and 52 weeks and at the end of follow-up yielded p-values of 0.0009, 0.004, 0.340, and 0.370, respectively. The median baseline CRP was 10 mg/L (mean: 21.7 + 5.8; IQR: 3-30; 95%CI: 9.8-33.6), which changed to 6.2 mg/L (mean: 14.0 + 3.2; IQR: 4-9; 95%CI: -9.2-37.3; p = 0.240) (Table 2).

**Table 2:** Clinical response and clinical remission at 12, 24, and 52 weeks and at the end of follow-up ESR: erythrocyte sedimentation rate; CRP: C-reactive protein; HBI: Harvey-Bradshaw index; IQR: interquartile range

Parameter	Baseline (n = 30)	Mean	IQR	12 weeks (n = 30)	Mean	IQR	p-value	24 weeks (n = 10)	Mean	IQR	p-value	52 weeks (n = 20)	Mean	IQR	p-value	End of follow-up (n = 6)	Mean	IQR	p-value
ESR	38	40.4	18-60	29	37.8	0-29	-	60	36.2	9-70	0.004	36	39.1	15-57	0.35	46	32.5	15-64	0.7
Parameter	Baseline (n = 29)	Mean	IQR	12 weeks (n = 30)	Mean	IQR	p-value	24 weeks (n = 12)	Mean	IQR	p-value	52 weeks (n = 22)	Mean	IQR	p-value	End of follow-up (n = 6)	Mean	IQR	p-value
CRP	10	21.7	3-30	5.7	14.6	3.3-22	0.23	9.61	16.7	1-25	0.48	5.9	12.2	3-17	0.10	6.2	14.0	4-9	0.24
Parameter	Baseline (n = 30)	Median	IQR	12 weeks (n = 26)	Median	IQR	p-value	24 weeks (n = 25)	Median	IQR	p-value	52 weeks (n = 27)	Median	IQR	p-value	At the end of follow-up (n = 11)	Median	IQR	p-value
HBI score		2	0-4		0	0-2	0.0009		2	0-2	0.004		0	0-2	0.34		0	0-0	0.37

Table 3 shows clinical and laboratory parameters at 12, 24, and 52 weeks and at the end of follow-up. Corticosteroid-free remission, clinical response, and clinical remission showed a decreasing trend in percentage between weeks 12 and 24 compared to week 52 where a spike was observed in all aforementioned parameters. The p-values from cross-tabulation were only significant for clinical response and clinical remission when comparing week 12 to weeks 24 and 52. The percentages at the end of follow-up were not significantly related to values at 12 weeks.

**Table 3:** Clinical and laboratory parameters at baseline and at 12, 24, and 52 weeks among ustekinumab-treated patients with Crohn’s disease

Parameters	12 weeks	24 weeks	p-value	52 weeks	p-value
Corticosteroid-free remission	23 (76.7)	23 (76.7)	0.104	26 (86.7)	0.58
Clinical response	22 (73.3)	21 (70)	0.001	23 (76.7)	0.05
Clinical remission	20 (66.7)	20 (66.7)	<0.0001	21 (70)	0.042
Any adverse events	-	1 (3.3)		-	

**Figure 1:**
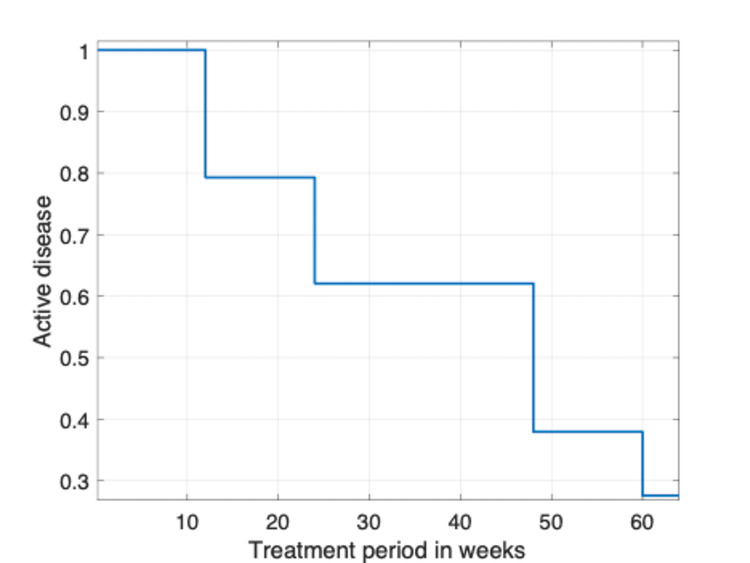
Remission rate during the treatment period

We used the Kaplan-Meier curve to investigate the trend of active disease during therapy (Figure 1). A clear reduction in active disease is seen. A careful analysis is required to interpret these results to determine the effectiveness of therapy.

Adverse event

No adverse events, such as malignancies or infections, were observed during the follow-up. However, only one patient had acne and rash symptoms at 24 weeks, unknown if related to UST and thus not warranting discontinuation of UST (Table 3).

## Discussion

This is a real-world study evaluating the short- and long-term data for the clinical effectiveness of UST retrospectively in a cohort of refractory CD patients from the Middle East. The study showed that the clinical remission ratios ranged from 66.7% and 70% in moderate to severely active CD patients at 12 and 52 weeks, respectively. The induction percentage was even higher for patients in the age group 17-40 years. The disease location was ileocolonic in most of the subjects. Most patients were treated with thiopurine and infliximab. In addition, 40% of the patients were in corticosteroid-free remission.

Hanauer et al. evaluated the induction treatment in the UNITI/IM-UNITI studies of UST among CD patients who entered a long-term extension (LTE) of up to five years post-induction [22]. Efficacy and safety profiles were evaluated through 152 and 156 weeks, respectively. At week 44 of IM-UNITI, 567 UST-treated CD patients entered long-term extension. These patients received blinded subcutaneous UST at their assigned dose interval, and no subsequent dose adjustments were made. Following this unblinding, the placebo-treated patients were discontinued. The efficacy data in LTE were collected every 12 weeks (q12w) preceding the study unblinding and then at q12w/q8w dosing visits. The results of this study suggested that 29.6% of UST-treated patients discontinued through week 156. The intent-to-treat analysis of randomized patients from IM-UNITI during the first 152 weeks indicated that 38% of UST induction responders receiving drug q12w and 43.0% q8w were in remission. Among the long-term extension patients from their original randomized groups, 61.9% of the q12w and 69.5% of the q8w patients were in remission by week 152. The remission rates were 56.3% and 55.1% for q12w and q8w, respectively, across all UST-treated patients (randomized and non-randomized) entering the long-term extension. In conclusion, continued subcutaneous UST treatment can effectively maintain the clinical response and remission for three years and is well tolerated [22].

Buckingham et al. conducted a retrospective analysis of the demographic characteristics, schedule, dosage, and medical history of patients treated with UST [23]. Furthermore, the data on pre- and post-ESR, calprotectin, and Pediatric Crohn’s Disease Activity Index (PCDAI) were also collected. In the study cohort, five patients were treated with UST with an age range of 8-15 years, with a male/female ratio of 3:2 and age of 2-10 years at the time of diagnosis. Of these, four patients were diagnosed with CD and one with UC. The subjects were followed up for up to 60 weeks post-treatment initiation. All five subjects had previously failed a minimum of two biologic treatments. All these patients received an initial single IV dose of 6 mg/kg UST over at least one hour and subsequent SC doses at eight weeks intervals, adjusted at 90 mg if >40 kg and 45 mg if <40 kg. In 80% of the subjects, UST significantly reduced ESR and calprotectin. Remarkable improvement was reported in the PCDAI and patient global assessment (PGA) scores for all the patients [23]. Our results are similar to other studies showing the effectiveness of ustekinumab in Crohn’s post-anti-TNF or vedolizumab failure [24,25].

Furthermore, Eberl et al. retrospectively explored real-life data in 48 Finnish CD patients receiving UST [26]. The study evaluated the efficacy profiles of various UST treatment patterns based on dosing frequency, persistence, and concomitant medication. Clinical remission and response rates were evaluated via a modified HBI (mHBI) and endoscopic response. The endoscopic response was assessed using a simple endoscopic score for CD (SES-CD) as the proportion of patients. Modified HBI and SES-CD were measured at week 16 and the end of follow-up [26]. Deepak and Loftus studied the immunological patterns associated with UST drug development through clinical trials [27]. The safety, efficacy, and pharmacokinetic profiles were evaluated to identify its potential place in the treatment of CD. The results suggest that 83% of the patients continued to receive UST treatment at the end of follow-up. The clinical response and endoscopic healing were evident during week 16, and mHBI reduced from 9 to 3 (p = 0.0001) and SES-CD from 12 to 3 (p = 0.009). Clinical benefit was estimated to be about 83% at week 16 and 76% at the end of follow-up. Another supporting evidence for UST was the reduction in the proportion of patients using corticosteroids from 48% at the start of the study to 25% at week 16 and 13% at the end of follow-up. The real-life nationwide research of CD patients concluded that UST could induce short-term clinical benefits and improve endoscopic response in a safe, effective, and persistent manner. Furthermore, significant corticosteroid tapering in highly treatment-refractory and long-standing CD patients was also reported [27].

This study has some limitations. First, this study has a retrospective design. Second, the outcome was mainly clinical and not based on a robust endoscopic response. Third, UST was probably continued without clinical response in some patients exposed to several biologics because there were no other therapeutic options. Fourth, the data on UST trough levels and antidrug antibodies were not available.

## Conclusions

This is the first study to show UST’s real-world efficacy and safety in a Middle Eastern cohort of moderate to severe CD patients. This study confirms UST’s effectiveness and safety in CD patients exposed to several anti-TNF agents. Overall, our CD patients’ demographic and clinical outcomes were comparable to those reported from other parts of the world.
